# Efficacy of a topical gabapentin gel in a cisplatin paradigm of chemotherapy-induced peripheral neuropathy

**DOI:** 10.1186/s40360-019-0329-3

**Published:** 2019-08-28

**Authors:** Muhammad Shahid, Fazal Subhan, Nisar Ahmad, Robert D. E. Sewell

**Affiliations:** 1 Department of Pharmacy, Sarhad University of Science and Information Technology, Peshawar, Pakistan; 2 Department of Pharmacy, University of Peshawar, Peshawar, 25120 Pakistan; 3 Department of Pharmacy, CECOS University, Hayatabad, Phase 6, Peshawar, Khyber Pakhtunkhwa Pakistan; 4 Department of Pharmacy, Abasyn University, Peshawar, Pakistan; 5 Cardiff School of Pharmacy and Pharmaceutical Sciences, Cardiff University, Cardiff, CF10 3NU UK

**Keywords:** Cisplatin, Neuropathic pain, Topical, Gabapentin, Topical gel, Allodynia and heat hypoalgesia

## Abstract

**Background:**

Chemotherapy induced peripheral neuropathy (CIPN) has been attributed to chemotherapeutic agents such as cisplatin which adversely affect disease outcome leading to increased cancer related morbidity. The clinical efficacy of systemic gabapentin in neuropathic pain management is limited by central side-effects in addition to a scarceness of conclusive evidence of its efficacy in CIPN management. The topical route therefore may provide a relatively safe alternative for neuropathic pain treatment in general and CIPN in particular.

**Methods:**

Cisplatin induced neuropathic nociception was established in rats after a single weekly cisplatin injection (3.0 mg/kg, intraperitoneally) for 4 weeks. The evoked neuropathic sensation of allodynia was assessed by plantar application of von Frey monofilaments as the paw withdrawal threshold (PWT), whereas the expression of heat-hypoalgesia was determined on a hot-plate as paw withdrawal latency (PWL). Gabapentin gel (10% w/w) was applied three-times daily on the hind paws while in a concurrent systemic study, gabapentin was administered daily (75 mg/kg, intraperitoneally) for 4 weeks. To assess any evidence of neurological adverse symptoms of cisplatin and the central side-effect propensity of systemic or topical gabapentin, evaluation of motor coordination (rotarod test) and gait (footprint analysis) were performed.

**Results:**

Cisplatin invoked a progressive development of neuropathic hind paw allodynia (decreased PWT, days 7–28) and heat hypoalgesia (increased PWL, days 21–28). Topical gabapentin significantly delayed the expression of both allodynia on protocol days 21 and 28 and heat-hypoalgesia (day 28). Systemic gabapentin displayed a comparative anti-neuropathic predisposition through a sustained suppression of tactile allodynia on days 14 and 21–28 as well as thermal hypoalgesia (days 21 and 28). Systemic gabapentin also impaired motor coordination and gait thus affirming its clinically documented central side effects, but these outcomes were not evident after topical treatment.

**Conclusions:**

Both topical and systemic gabapentin exhibit a propensity to attenuate CIPN in a cisplatin paradigm. Gabapentin applied topically may therefore provide an adjunctive or alternative route for CIPN management upon cessation of systemic medications due to intolerable side-effects.

**Electronic supplementary material:**

The online version of this article (10.1186/s40360-019-0329-3) contains supplementary material, which is available to authorized users.

## Background

Chemotherapy induced peripheral neuropathy (CIPN) is a frequent pathological complication in patients undergoing cancer treatment. This condition involves sensory symptoms typically in the hands and feet and may include pain, numbness or tingling and motor symptoms expressed as weakness. It not only affects the patient’s response to treatment due to the need for dose reduction or discontinuation, but also there may well be a long term disruption of quality of life [1]. The prevalence of CIPN differs with regard to the type of chemotherapeutic agent used (72.3% with oxaliplatin, 42.2% with cisplatin, 70.8% with paclitaxel, 19.6% with vincristine, 63.5% with thalidomide, and 46.7% with bortezomib), and the duration of chemotherapy (68.1% in the first month, 60.0% at 3 months and 30.0% at 6 months) [2]. Additionally, the presence of CIPN causes gait disturbances and may increase the risk of falls in cancer patients undergoing chemotherapy [3, 4].

Cisplatin is a platinum-based drug which is highly effective against various types of cancers, including carcinomas, germ cell tumors, sarcomas and lymphomas [5]. In addition, cisplatin has established the highest cure rates in the management of testicular cancers (90%) [6, 7]. Although cisplatin has been considered a mainstay treatment for cancer, its use is restricted by the induction of resistance to its beneficial effects in cancer cells and the occurrence of side-effects including nausea and vomiting, neurotoxicity, ototoxicity, and renal injury [6, 8–11]. Many patients completing a full course of cisplatin chemotherapy develop a clinically detectable sensory neuropathy. These symptoms include unpleasant distal paresthesias (tingling in the extremities) and numbness that may occur as soon as a month after initiating treatment, Lhermitte’s symptom (an electric shock-like sensation on bending the neck), indicating the involvement of the centripetal branch of the sensory pathway within the spinal cord, large fiber sensory loss (reduced vibration and joint position sensations) and diminished or absent muscle stretch reflexes, sensory ataxia (incoordination) and mildly diminished small fiber sensation (decreased pin-pain sensation). These neuropathic symptoms are a major reason for the premature discontinuation of cisplatin and limitation of its cumulative dosage, thereby potentially reducing its chemotherapeutic efficacy [12].

The pharmacotherapy of systemic disorders including peripheral neuropathy is a challenging task for clinicians as well as biomedical scientists and recently, various therapeutic moieties have been investigated for their beneficial effects in neuropathic pain [13–19]. Different treatment modalities have been devised for platinum induced neuropathy which includes neuroprotective agents, antidepressants and anticonvulsants [1, 12, 20]. Despite these preventive and therapeutic strategies, treatment modification and drug withdrawal remain the most effective modalities for a majority of patients with CIPN. However, further preclinical and clinical research is needed to establish better alternative options [20]. The gabapentinoid, gabapentin has been one of the first line drugs used in clinical practice for the treatment of patients with established neuropathy. Preclinical studies have demonstrated that gabapentin is able to attenuate both the positive and negative neuropathic symptoms of CIPN [15, 21]. However, there is conflicting evidence of gabapentin efficacy in clinical trials, with some studies showing a meaningful reduction in pain scores in patients [22, 23], while others have obtained negative results [24, 25]. Irrespective of these observations, gabapentin has been considered a common choice of clinicians to manage the positive symptoms associated with CIPN [26, 27]. However, the therapeutic efficacy of anti-neuropathic doses of gabapentin is greatly hindered by side-effects such as dizziness, somnolence, ataxia, weight gain, lethargy, and convulsions [28–31]. The occurrence of side-effects along with therapy specific precautions and contraindications has limited the clinical analgesic utility of the current pharmacological treatments and only < 50% of neuropathic patients actually show any improvement in their pain states [30].

There is a recent trend for targeting the peripheral nervous system in neuropathic pain and from this perspective, various topical agents have been compounded and successfully tested in patients and various animal models [32–34]. Nociceptors in layers of the skin contain various types of receptor that bind different ligands which influence the generation of pain transmitting action potentials. Topical formulations traverse epidermal tissue and increase the nociceptive threshold by stabilizing the membranes of specific nociceptors [35]. The topical route presents distinct advantages because there is low systemic clearance, minimum chance of drug interaction, relative patient tolerability and the feasibility of combination with various oral medications [36]. A variety of topical preparations have been investigated in CIPN. These include a baclofen with amitriptyline plus ketamine organogel (BAK) combination [37], low-concentration menthol [38, 39], phenytoin [40], an amitriptyline and ketamine cream [41] and topical combinations of α_2_-adrenergic receptor agonists or nitric oxide (NO) donors combined with either phosphodiesterase (PDE) or phosphatidic acid (PA) inhibitors [42].

Considering the beneficial anti-neuropathic profile of systemic gabapentin, its central side-effect tendency and the inconsistency of its effectiveness in CIPN, this study investigated the possible efficacy of a gabapentin (10%) topical gel formulation in a refined CIPN rat model of peripheral neuropathic pain. Previously, this gel has been reported to alleviate both mechanical allodynia and vulvodynia in an animal model of streptozotocin-induced diabetic neuropathic nociception [33] and in the traumatic nerve injury model of neuropathic allodynia and hyperalgesia [43].

## Methods

### Chemicals

Topical gabapentin as a 10% w/w gel and the control gel base (an oil in water gel comprising xanthan gum hydrocolloid with polyacrylamide minus the active pharmaceutical ingredient) [supplied by St Mary’s Pharmaceutical Unit (SMPU, Cardiff, UK under their Manufacturer’s Special License (MSL)], gabapentin active (99.53% was obtained from Lowitt Pharmaceuticals, Peshawar, Pakistan). Both cisplatin and gabapentin were dissolved in normal saline.

### Animals

Male Sprague Dawley rats (200–250 g) were bred at the animal house facility in the Department of Pharmacy, University of Peshawar, Peshawar, Pakistan. They were maintained in a 12 h/12 h light/dark cycle at 22 ± 2 °C with ad libitum access to food and water. The experimental procedures on animals were performed in compliance with the UK Animals (Scientific Procedures) Act 1986 and according to the rules and ethics set forth by the Institutional Ethical Committee. Approval for the study was granted from the Ethical Committee of the Department of Pharmacy, University of Peshawar with the registration number: 13/EC-15/Pharm. At the end of experiments, the animals were euthanized by cervical dislocation under anesthesia (intraperitoneal injection of a mixture of xylazine at 10 mg/kg and ketamine at 100 mg/kg).

### Cisplatin induced neuropathy treatment schedule

Cisplatin-induced neuropathic nociception was established using four cisplatin intraperitoneal injections (3.0 mg/kg) each at weekly intervals as previously reported [21]. Before each cisplatin injection, hyperhydration was induced by the subcutaneous injection of 2.0 mL normal saline in order to avoid cisplatin associated nephrotoxicity. The hydration strategy has been shown to significantly lower the incidence of cisplatin-induced renal damage [44] and has been recommended for preventing cisplatin-induced nephrotoxicity before and after administration of cisplatin [45, 46].

To assess the effect of topical gabapentin, a uniform quantity of 10% gabapentin gel (1.0 mg/cm^2^) (GBP-10%) was applied topically three times daily on the plantar surface of both hind paws [43]. An equivalent amount of control gel (1.0 mg/cm^2^) (CG) was also applied on the hind paws of the control animals. In the systemic study, gabapentin was administered intraperitoneally once daily at a dose of 75 mg/kg (GBP-75) [33]. Moreover, as a negative control, a group of cisplatin injected animals were systemically administered (i.p.) once daily with equal volume of saline. The animals were randomly assigned to the following treatment groups (*n* = 6) and the study was continued for 28 days:

- Group 1: Saline (1.0 mL/kg, i.p.)
- Group 2: Cisplatin (3.0 mg/kg/week, i.p.)
- Group 3: Saline (1.0 mL/kg, i.p.) + Cisplatin (3.0 mg/kg/week, i.p.)
- Group 4: GBP-10% (1.0 mg/cm^2^/× 3/day) + Cisplatin (3.0 mg/kg/week, i.p.)
- Group 5: GBP-75 (75 mg/kg/day, i.p.) + Cisplatin (3.0 mg/kg/week, i.p.)
- Group 6: CG (1.0 mg/cm^2^/× 3/day) + Cisplatin (3.0 mg/kg/week, i.p.)
- Group 7: GBP-10% (1.0 mg/cm^2^/× 3/day)
- Group 8: GBP-75 (75 mg/kg/day, i.p.)
- Group 9: CG (1.0 mg/cm^2^/× 3/day)

### Neuropathic paradigm of static allodynia

The mechanical allodynia expression in the hind paws after dosing with cisplatin was evaluated using von Frey filaments (Stoelting, Wood Dale, Illinois, USA). Each filament was applied to the mid-plantar surface until it buckles [47] and the nociceptive response was measured as the paw withdrawal threshold (PWT, g). The static allodynia paradigm was determined at the end of each week [13, 48].

### Neuropathic paradigm of heat hypoalgesia

The expression of cisplatin associated heat hypoalgesia was assessed in rat hind paws using a digital hot-plate apparatus (Harvard apparatus, USA). The hot-plate was thermostatically maintained at 52.0 ± 0*.*2 °C and the escape phenomenon was measured in seconds as jumping or hind paw lifting/licking. A cut-off time limit of 40 s was selected in order to avoid tissue injury. Each response latency was quantified as the paw withdrawal latency (PWL). Neuropathic heat-hypoalgesia was measured at the end of each week of the 4 weeks’ paradigm [13].

### Motor coordination and balance

The motor coordination and the central side-effects propensity was assessed using the rotarod paradigm. Each animal was placed on a variable speed rotating rod and the endurance latency was determined in seconds on days 7, 14, 21 and 28 after 60–80 min post topical or systemic treatment [43]. The gait impairment was evaluated using footprint analysis. The overlap between forepaw and hind paw placement was quantified 60–80 min post topical or systemic treatment [43].

### Data analysis

The data were presented as mean ± standard error of the mean (SEM) and were analyzed by two-sample *t*-test (saline and cisplatin only groups) or by two-way repeated measures analysis of variance (ANOVA) followed by post hoc Bonferroni’s test (cisplatin injected groups treated with saline and systemic or topical gabapentin). The neuropathic paradigms of paw withdrawal thresholds and latencies were converted into percentage anti-allodynia and heat anti-hypoalgesia, respectively. Moreover, the rotarod dismount latencies and the paw overlap distance were respectively transformed into motor incoordination and paw displacement indices as previously reported [13]. All statistical analyses were conducted using GraphPad Prism 5 (GraphPad Software Inc. San Diego CA, USA). A value of *P* < 0.05 was considered as significant.

## Results

### Topical gabapentin gel de-escalation of cisplatin neuropathic allodynia

**Fig. 1:**
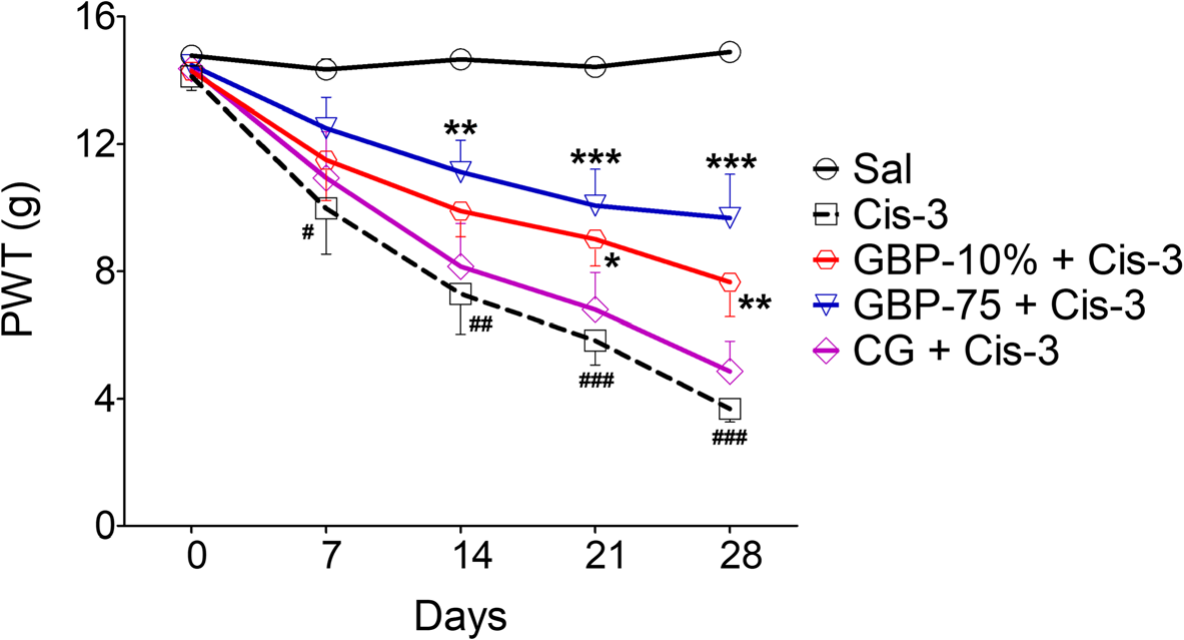
Effect of topical gabapentin 10% gel (GBP-10%), topical control gel (CG) and systemic gabapentin at 75 mg/kg (GBP-75, i.p.) on the expression of cisplatin induced static allodynia [diminished von Frey filament threshold pressure (paw withdrawal threshold; PWT in g)] in hind paws after weekly intraperitoneal injection of cisplatin at 3.0 mg/kg (Cis-3) for four consecutive weeks in rats. Each symbol represents the mean PWT in g ± SEM. ^#^*P* < 0.05, ^##^*P* < 0.01, ^###^*P* < 0.001 as compared to the saline (Sal) alone treated control group, ^*^P < 0.05, ^**^P < 0.01, ^***^P < 0.001 as compared to the cisplatin plus saline treated group, two-way repeated measures ANOVA followed by post hoc Bonferroni’s analysis; *n* = 6 rats per group

Single weekly administration of cisplatin was associated with a marked decrease in the threshold to perceive nociception evoked by non-nociceptive static stimuli. This was evident from a tapering decline of nociceptive thresholds with cumulative cisplatin doses. The application of graded von Frey filaments against the mid-plantar surface of the hind paws elicited a response observed as a brisk paw withdrawal. This aberrant behavior was detected as a significant decrease in the applied force in grams and was primarily noted after the first week of cisplatin injection [day 7: 9.97 g; t(5) = 2.760, *P* = 0.0398] as compared to that of the saline treated controls (14.33 g on day 7). After the first week, a temporal decrement in threshold was apparent as a graded reduction in nociception to the normally non-nociceptive stimulus. Thus a significant decrease in the threshold paw withdrawal response was expressed to the increasing static pressure of von Frey filaments in the cisplatin dosed animals in the 2nd week [day 14: 7.28 g; t(5) = 5.602, *P* = 0.0025] and 3rd week of the study [day 21: 5.82 g; t(5) = 12.34, *P* < 0.0001]. A fully expressed static allodynia emerged after the last cisplatin injection (4th week) during which the reduction in the nociceptive threshold was fully discernable [day 28: 3.68 g; t(5) = 26.25, *P* < 0.0001]. Similarly, the saline treated cisplatin animals also presented with a similar manifestation of static hind paw allodynia after week 1 [day 7: 10.3 g; t(5) = 2.722, *P* = 0.0417], week 2 [day 14: 7.75 g; t(5) = 4.853, *P* = 0.0047], week 3 [day 21: 5.38 g; t(5) = 9.597, *P* = 0.0002], and week 4 [day 28: 3.76 g; t(5) = 23.73, *P* < 0.0001] as compared to the respective thresholds of the saline treated non-cisplatin dosed control animals (14.66 g on day 14, 14.42 g on day 21, and 14.88 g on day 28) (Fig. 1 and Additional file 1: Table S1).

Treatment with topical as well as systemic gabapentin produced a beneficial antinociceptive action against the expression of cisplatin induced hind paw allodynia [time = (*F* (4, 200) = 52.75, *P* < 0.0001), treatment = (*F* (8, 200) = 65.01, *P* < 0.0001), interaction = (*F* (32, 200) = 5.87, *P* < 0.0001)]. When the gel formulation of gabapentin (10%) was applied topically three times daily on the hind paw plantar surface, an elevated paw withdrawal threshold was observed which reversed the cisplatin induced progressive decline in the nociceptive threshold. It was notable that the allodynia offsetting effect of gabapentin gel was not evident after 14 days (2nd injection of cisplatin). However, a distinguishable increase in the diminished neuropathic threshold force was noticeable after the 3rd cisplatin injection when a significant increase in PWT (day 21: 9.01 g, *P* < 0.05) was observed as compared to the cisplatin untreated animal group. This effect became more pronounced after the 4th and last cisplatin injection (day 28: 7.65 g, *P* < 0.01). The concurrent topical application of the control gel was devoid of any cisplatin allodynia modifying action throughout the four-week testing paradigm (6.79–4.86 g on days 14–21). Systemic administration of gabapentin (75 mg/kg) reversed the cisplatin-downgraded threshold and it was more effective in this respect than the topical route. The increase in perceived static force was found to be significant after the second cisplatin injection (11.12 g, *P* < 0.01) and this was maintained for the subsequent study period i.e. after the 3rd week (10.06 g, *P* < 0.001) and 4th week (9.67 g, *P* < 0.001) of the cisplatin injection protocol (Fig. 1 and Additional file 1: Table S1).

The antinociceptive activity of topical and systemic gabapentin against cisplatin neuropathic allodynia was substantiated by our findings [time = (*F* (3, 120) = 0.94, *P* = 0.6913), treatment = (*F* (6, 120) = 65.10, *P* < 0.0001), interaction = (*F* (18, 120) = 0.94, *P* = 0.5297)]. Thus, a significant increase in percentage antinociception was afforded by topical gabapentin on day 21 (37.51%, *P* < 0.05) and day 28 (38.08%, *P* < 0.05), as compared to the cisplatin administered saline treated group (− 4.89 and 0.79%). The percentage protection provided by systemic gabapentin was found to be greater, disclosing a significant increase on test day 14 (48.78%, *P* < 0.05), day 21 (51.99%, *P* < 0.001), and day 28 (53.27%, *P* < 0.01). The inefficacy of the control gel in attenuating the neuropathic symptoms was endorsed by non-significant intergroup anti-allodynic differences compared to the saline treated cisplatin animals (16.69 and 5.47% on days 21–28). No deviant threshold changes indicative of any allodynic tendency were observed in the groups of animals treated alone with gabapentin 10% gel, control gel and systemic gabapentin at 75 mg/kg. Additionally, the responses of these groups were found to be significantly different from the cisplatin untreated or saline-treated animals in terms of the pressure required to elicit a response (*P* < 0.001) or percentage protection (*P* < 0.001).

### Topical gabapentin gel alleviation of cisplatin neuropathic hypoalgesia

**Fig. 2:**
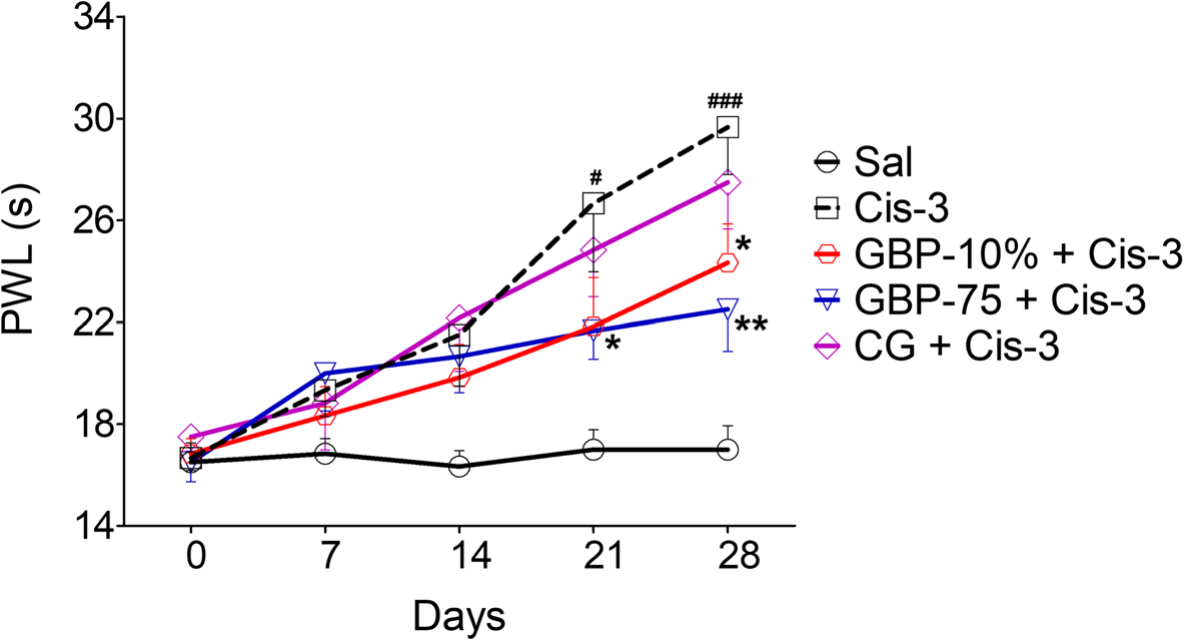
Effect of topical gabapentin 10% gel (GBP-10%), topical control gel (CG) and systemic gabapentin at 75 mg/kg (GBP-75, i.p.) on the expression of cisplatin induced heat hypoalgesia [increased nociceptive response latency to heat stimulus in the hot-plate paradigm (paw withdrawal latency; PWL in s)] in bilateral hind paws after weekly intraperitoneal injection of cisplatin at 3.0 mg/kg (Cis-3) for four consecutive weeks. Each symbol represents the mean PWL in s ± SEM. ^#^*P* < 0.05, ^###^*P* < 0.001 as compared to the saline (Sal) treated controls, ^*^P < 0.05, ^**^P < 0.01, as compared to the cisplatin plus saline treated animal group, two-way repeated measures ANOVA followed by post hoc Bonferroni’s analysis; *n* = 6 rats per group

Intraperitoneal administration of cisplatin impaired the perception of the heat stimulus on the hot plate. This cisplatin induced neuropathic thermal hypoalgesia followed a slower course of onset because there was no significant increase in paw withdrawal after the second weekly cisplatin dose [day 14: 21.50 s; t(5) = 2.260, *P* = 0.0734]. However, following the 3rd injection of cisplatin, a significant difference in the thermal withdrawal latency was noted between the cisplatin administered animals and the saline treated control group. Hence, there was a marked increase in the paw thermal threshold on protocol day 21 [26.67 s; t(5) = 3.932, *P* = 0.0110] and this thermal hypoalgesia was maintained with an increased intensity after the last cisplatin injection i.e. at the end of the 4th week [day 28: 29.67 s; t(5) = 7.340, *P* = 0.0007]. The cisplatin treated group which received saline, also exhibited a similar thermal hypoalgesia expression profile with an increased hot-plate latency being detected after week 2 [day 14: 21.67 s; t(5) = 2.769, *P* = 0.0394] and this effect was even more pronounced at the end of week 3 [day 21: 25.83 s; t(5) = 3.466, *P* = 0.0179], and week 4 [day 28: 29.17 s; t(5) = 3.720, *P* = 0.0137] of the cisplatin injection protocol (Fig. 2 and Additional file 1: Table S2).

The three times daily topical application or daily systemic intraperitoneal administration of gabapentin offset the expression of thermal hypoalgesia in the hind paws [time = (*F* (4, 200) = 33.10, *P* < 0.0001), treatment = (*F* (8, 200) = 17.17, *P* < 0.0001), interaction = (*F* (32, 200) = 2.42, *P* = 0.0001)]. Topical gabapentin (10%) gel reversed the elevated neuropathic thermal paw reaction latencies by the end of the third week (day 21: 21.83 s) and this was more marked at the end of the fourth week (day 28: 24.33 s, *P* < 0.05). There was no significant change in paw reaction latencies in the control gel treated animals compared to the cisplatin alone group, but systemic gabapentin did reduce withdrawal responses associated with cisplatin treatment. Accordingly, a significant attenuation of the cisplatin prolonged PWL was apparent by test day 21 of the paradigm (21.66 s, *P* < 0.05) and day 28 (22.50 s, *P* < 0.01). In contrast and somewhat predictably, the group of animals treated with saline was not found to induce any detectable alteration of cisplatin heat hypoalgesia at any time during the whole period of the four-week protocol (Fig. 2 and Additional file 1: Table S2).

The anti-neuropathic hypoalgesic efficacy underlying the topical and systemic gabapentin hot-plate response was confirmed by our findings [time = (*F* (3, 120) = 4.54, *P* = 0.0139), treatment = (*F* (6, 120) = 20.54, *P* < 0.0001), interaction = (*F* (18, 120) = 2.51, *P* = 0.0016)]. Protection provided by the gabapentin gel formulation was more clear-cut after protocol week 4 when the latency difference achieved statistical significance (day 28: -19.93%, *P* < 0.01). Likewise, systemic gabapentin also produced a similar heat-hypoalgesia attenuating trend by producing a non-significant percentage neuropathic hypoalgesia aberration on day 21 (− 13.84%) followed by a significant suppressive effect on study day 28 (− 26.02%, *P* < 0.001) whereas the control gel was inactive throughout.

### Motor discoordination induced by systemic but not topical gabapentin

**Fig. 3:**
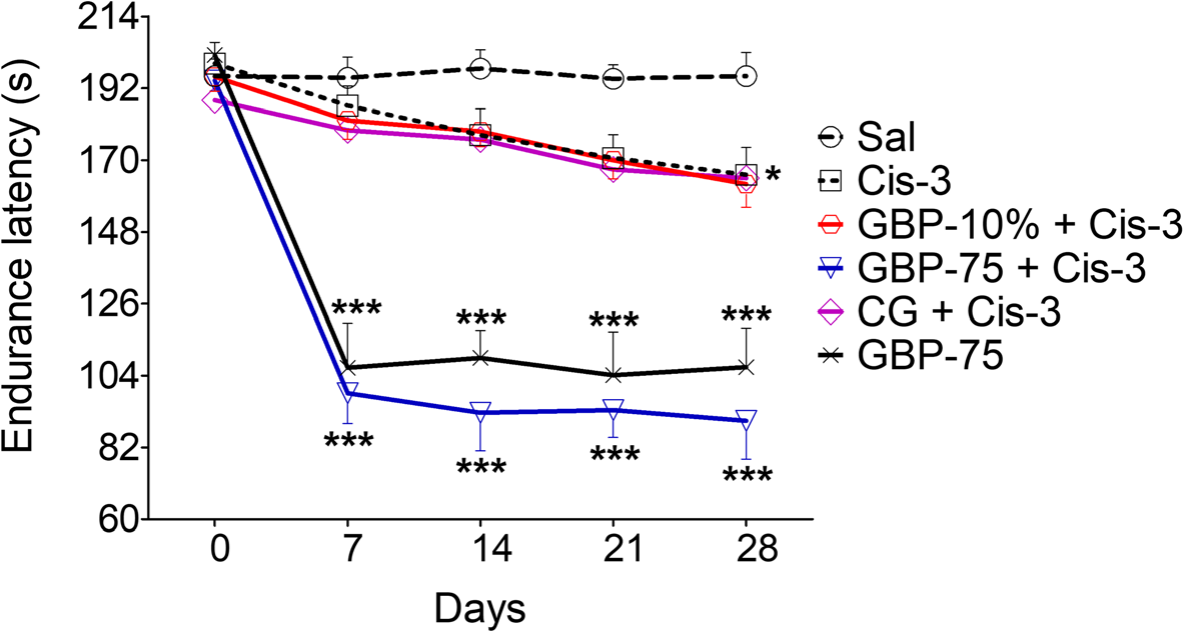
Effect of topical gabapentin 10% gel (GBP-10%), topical control gel (CG) and systemic gabapentin at 75 mg/kg (GBP-75, i.p.) on rotarod performance after weekly i.p. injection of cisplatin (Cis) at 3.0 mg/kg (Cis-3) for four consecutive weeks in rats. Each symbol represents the mean endurance latency in s ± SEM, 1 h post treatment. ^*^P < 0.01, ^***^P < 0.001 as compared to the saline (Sal) treated control group, two-way repeated measures ANOVA followed by post hoc Bonferroni’s analysis; *n* = 6 rats per group

Considerable changes in motor coordination and locomotor gait were observed in the rotarod and footprint analysis paradigms after administration of cisplatin and gabapentin [time = (*F* (4, 200) = 32.14, *P* < 0.0001), treatment = (*F* (8, 200) = 101.33, *P* < 0.0001), interaction = (*F* (32, 200) = 6.89, *P* < 0.0001)]. A progressive decline in endurance latency on the accelerating rotarod was observed in the groups of animals administered cisplatin either alone or along with saline. There was no significant reduction noted at the end of week 2 (day 14: 177.6 s) and week 3 (day 21: 170.6 s), but a significant deterioration in endurance latency after the final cisplatin injection i.e. week 4 (165.5 s, *P* < 0.05). A marked impairment of motor coordination was detected in the cisplatin plus systemic gabapentin (75 mg/kg) treated animal group as well as those administered systemic gabapentin alone. Thus, the cisplatin dosed animals co-treated with systemic gabapentin exhibited a significant decrease in rotarod endurance latency on paradigm day 7 (98.66 s, *P* < 0.001), day 14 (92.66 s, *P* < 0.001), day 21 (93.50 s, *P* < 0.001) as well as day 28 (90.16, *P* < 0.001). Similarly, treatment with systemic gabapentin by itself was also associated with a significant decrease in dismount latency on day 7 (106.50s, *P* < 0.001), day 14 (109.50s, *P* < 0.001), day 21 (104.16 s, *P* < 0.001) and day 28 (106.66 s, *P* < 0.001), in comparison with corresponding saline controls (Fig. 3 and Additional file 1: Table S3).

The rotarod endurance latency [time = (*F* (3, 160) = 2.40, *P* = 0.0981), treatment = (*F* (8, 160) = 336.30.43, *P* < 0.0001), interaction = (*F* (24, 160) = 1.18, *P* = 0.2694)] also disclosed a significant motor impairment in the animals administered cisplatin on its own (day 14: 10.43%, *P* < 0.05; day 21: 12.53%, *P* < 0.05; day 28: 15.52%, *P* < 0.001), and in the group given cisplatin plus injected saline (day 14: 10.33%, *P* < 0.05; day 21:12.66%, *P* < 0.05; day 28: 14.10%, *P* < 0.01) as compared to the saline alone treated control group (1.62, 3.72, 2.71% during days 14–28). Motor discoordination was also exhibited in the groups of cisplatin administered animals treated with topical gabapentin gel (9.61, 12.8%, *P* < 0.05 during days 14–21; and 16.8%, *P* < 0.001 on day 28) as well as the control gel (10.80%, *P* < 0.05 on day 14; 14.30%, *P* < 0.01 on day 21; and 15.88%, *P* < 0.001 on day 28). An extensive deficit in motor coordination (*P* < 0.001) was created by systemic gabapentin treatment on its own as well as in combination with cisplatin (day 7: 49.83 and 46.09%, day 14: 53.77 and 45.03%, day 21: 52.27 and 47.07%, and day 28: 54.53 and 46.20%), as compared to saline treated controls.

**Fig. 4:**
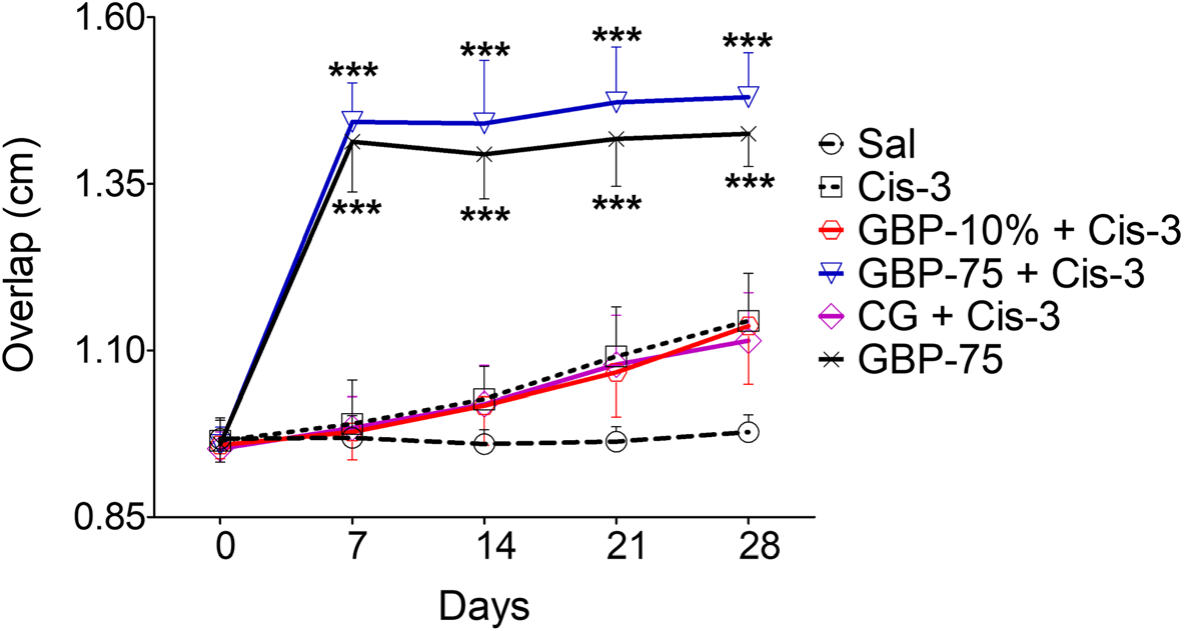
Effect of topical gabapentin 10% gel (GBP-10%), topical control gel (CG) and systemic gabapentin at 75 mg/kg (GBP-75, i.p.) on footprint pattern analysis after weekly i.p. injection of cisplatin (Cis) at 3.0 mg/kg (Cis-3) for four consecutive weeks in rats. The measured parameter was expressed as the overlap distance between the forepaw and hind paw placements, 1 h post treatment. Each symbol represents the mean paw overlap in cm ± SEM. ^***^P < 0.001 as compared to the saline (Sal) treated control group, two-way repeated measures ANOVA followed by post hoc Bonferroni’s analysis; *n* = 6 rats per group

Footprint pattern analysis revealed that the animals systemically treated with gabapentin expressed a marked disturbance of locomotor gait and this was thought to be derived from a disruption of balance [time = (*F* (4, 200) = 8.66, *P* = 0.0002), treatment = (*F* (8, 200) = 47.42, *P* < 0.0001), interaction = (*F* (32, 200) = 3.31, *P* < 0.0001)]. This major unwanted effect was recorded as a significant increase (*P* < 0.001) in the overlap distance between the forepaw and hind paw placements after treatment with gabapentin in the cisplatin injected animals in addition to those administered gabapentin by itself during the entire study period i.e. day 7 (1.44 cm and 1.41 cm), day 14 (1.44 cm and 1.39 cm), day 21 (1.47 cm and 1.42 cm), and day 28 (1.48 cm and 1.42 cm) as compared to the saline controls on the appropriate corresponding days (0.97 cm on day 7, 0.95 cm on day 14, 0.96 cm on day 21, and 0.98 cm on day 28). Additionally, there was no significant disturbance of locomotor gait produced in the cisplatin administered animals or by saline treatment alone, particularly at the end of week 3 (1.09 cm and 1.07 cm on day 21) and week 4 (1.14 cm and 1.13 cm on day 28) as shown in Fig. 4 and Additional file 1: Table S4.

The percentage foot displacement [time = (*F* (3, 160) = 0.97, *P* = 0.4260), treatment = (*F* (8, 160) = 241.26, *P* < 0.0001), interaction = (*F* (24, 160) = 1.63, *P* = 0.0406)] further substantiated the significant degree of walking impairment (*P* < 0.001) after treatment with systemic gabapentin (75 mg/kg) by itself or in combination with cisplatin on protocol day 7 (− 48.82% and − 45.41%), day 14 (− 49.43% and − 44.95%), day 21 (− 52.43% and − 46.73%), and day 28 (− 51.07% and − 45.75%), as compared to that of the respective saline group (− 1.03, 2.11, 1.01 and 0.51%). In addition to this, a significant impairment of balance was also observed in the cisplatin untreated and saline treated animals (day 21: -12.76%, *P* < 0.001 and − 11.40%, *P* < 0.01; and *P* < 0.001 on day 28: − 16.50% and − 15.78%) as compared to the saline controls. There was no significant distortion of gait by cisplatin, topical gabapentin or control gel treatment up to protocol day 14 though there was subsequent mild gait disturbance by cisplatin and topical gabapentin which attained statistical significance (*P* < 0.01 on day 21: − 10.34% and − 11.46%, and *P* < 0.001 on day 28: − 15.52% and − 13.48%). Moreover, topical gabapentin and the control gel were not associated with any variation in foot placement during the entire study.

## Discussion

The present study evaluated the antinociceptive effectiveness of topical gabapentin gel in relation to systemic gabapentin in a well-established cisplatin rat model of CIPN. Cisplatin administration has been reported to be associated with the development of mechanical allodynia and hyperalgesia, cold thermal allodynia and hyperalgesia, as well as heat thermal hypoalgesia in rats [21, 49]. Although in humans, most anticancer chemotherapeutic drugs are given intravenously, especially by continuous intravenous infusion, in this study, the peripheral neuropathic pain conditions associated with cisplatin was established by intraperitoneal injection of cisplatin. It has been observed in rats that the cisplatin levels in intra-abdominal tumor tissues following the use of the intraperitoneal route are greater than those after the intravenous route. In addition, concentrations of cisplatin in serum are also sufficiently greater with prolonged drug elimination after the use of the intraperitoneal route [50]. In another study, cisplatin injected intraperitoneally, significantly prolonged the lifespan median by 88% and in fact, it was ineffective when injected intravenously, in experimental rats with disseminated ovarian cancer [51]. Moreover, it has been confirmed clinically that intraperitoneal chemotherapy is feasible with acceptable toxicity and that intraperitoneal compared with intravenous cisplatin combination therapy yields a slight improvement in progression-free survival and overall survival of optimally cytoreduced advanced ovarian cancer [52].

This study has shown that, similar to systemic treatment, daily topical application of gabapentin strongly attenuated cisplatin associated neuropathic allodynia and heat-hypoalgesia. Topical application of 10% gabapentin caused a substantial reduction of pain in patients afflicted with different types of neuropathy [53, 54]. Compounded formulations containing gabapentin (6%) in combination with other drugs are effective in relieving pain conditions in > 75% of patients with neuropathies [55]. Moreover, topical 10% gabapentin has also been shown to allay both static and dynamic allodynia as well as vulvodynia in an animal model of streptozotocin induced polyneuropathy [33]. It also produced effectiveness against chronic constriction injury of the sciatic nerve induced tactile as well as cold-allodynia, along with heat and mechanical hyperalgesia in a rodent model of mononeuropathy [43]. Hence, these studies corroborated the antinociceptive efficacy of the topical formulation of gabapentin in neuropathic pain.

The value of the skin as a target for topical as well as the systemic medications to treat neuropathic pain is supported by studies demonstrating the importance of skin as a neuroimmunocutaneous system [32, 56]. Topical formulations traverse epidermal tissue and increase the nociceptive threshold by stabilizing the membranes of specific nociceptors including α_2_-adrenergic receptors, NMDA receptors, TRPVI receptors, and sodium channels [35]. Furthermore, the different types of non-neural cells in the skin also contain a variety of ion channels like those for Na^+^ and pharmacological receptors such as vanilloid, neurokinin, serotonin, cannabinoid, NMDA and GABA_A_ receptors that may be modified by topical drugs to regulate communication with dermal neurons to elicit robust analgesia [32]. It is decidedly possible therefore; that these mechanisms might be responsible for the antinociceptive effect of topical gabapentin in cisplatin induced neuropathic nociception.

In this study, a convincing relieving outcome on cisplatin-induced heat hypoalgesia was perceptible for daily systemic treatment with gabapentin only on expression days 21 and 28. This lesser efficacy of systemic gabapentin on cisplatin-induced heat hypoalgesia can be attributed to the inherent antinociceptive propensity of gabapentin. Systemic gabapentin has a central antinociceptive activity and is able to attenuate acute phasic thermal nociception [57, 58]. The pharmacological effects of gabapentin operate by: increasing GABA levels [59], acting as a non-NMDA receptor antagonist [60, 61], inhibiting voltage gated calcium channels [62, 63], inhibiting anterograde axoplasmic transport of α_2_δ-1 subunits, decreasing the release of glutamate, CGRP and substance P, decreasing microglial activation, reducing the number of astrocytes and inhibiting protein kinase C as well as TRP ion channels [64].

Systemic treatment with gabapentin in clinical doses adjusted for neuropathic pain has been associated with somnolence, dizziness, ataxia, and fatigue [28]. This can be further endorsed by the withdrawal rate of patients due to such adverse effects in clinical studies on systemic gabapentin efficacy in CIPN [23]. In this study, using different testing paradigms, systemic gabapentin produced only a transient increase in locomotion but induced considerable impairment of motor coordination. Ostensibly, topical gabapentin gel has not been associated with any of these unwanted systemic effects. The occurrence of side effects, along with therapy specific precautions and contraindications has limited the clinical utility of pharmacological treatments and only < 50% of neuropathic patients actually show any improvement in their pain states [30]. Although the systemic pharmacotherapy of painful neuropathy is currently the standard treatment approach, given the concomitant side effects, limited response rates, and potential for drug interactions, the use of the topical route may be a useful option for the effective as well as safe management of neuropathic pain [65, 66].

## Conclusion

The topical and systemic administration of gabapentin diminished chemotherapy associated peripheral neuropathic-like pain. There was as a significant attenuation of neuropathic allodynia and heat-hypoalgesia observed in a refined cisplatin rodent model of CIPN. The findings suggest that gabapentin has the potential to address the unmet pain reducing needs of patients diagnosed with chronic neuropathic pain undergoing treatment with chemotherapeutic drugs like cisplatin. In addition, it may also provide an alternative option for alleviation of neuropathic pain in the form of a compounded topical formulation if systemic medications are stopped due to intolerable side effects. Consequently, this delivery route for gabapentin may also be utilized as part of a comprehensive multi-mode pain management system. Definitive confirmation of this proposition can only be achieved if tested clinically in cancer patients suffering neuropathic pain.

## Additional file

Additional file 1: **Table S1.** Effect of per se treated topical gabapentin 10% gel (GBP-10%), topical control gel (CG) and systemic gabapentin at 75 mg/kg (GBP-75, i.p.) during the expression of cisplatin-induced static allodynia [diminished von Frey filament threshold pressure (paw withdrawal threshold; PWT in g)] in hind paws after weekly intraperitoneal injection of cisplatin at 3.0 mg/kg (Cis-3) for four consecutive weeks in rats. **Table S2.** Effect of per se treated topical gabapentin 10% gel (GBP-10%), topical control gel (CG) and systemic gabapentin at 75 mg/kg (GBP-75, i.p.) during the expression of cisplatin-induced heat hypoalgesia [increased nociceptive response latency to heat stimulus in the hot-plate paradigm (paw withdrawal latency; PWL in s)] in bilateral hind paws after weekly intraperitoneal injection of cisplatin at 3.0 mg/kg (Cis-3) for four consecutive weeks in rats. **Table S3.** Effect of per se treated topical gabapentin 10% gel (GBP-10%), topical control gel (CG) and systemic gabapentin at 75 mg/kg (GBP-75, i.p.) on rotarod performance during neuropathic nociception induced after weekly intraperitoneal injection of cisplatin (Cis) at 3.0 mg/kg (Cis-3) for four consecutive weeks in rats. **Table S4.** Effect of per se treated topical gabapentin 10% gel (GBP-10%), topical control gel (CG) and systemic gabapentin at 75 mg/kg (GBP-75, i.p.) on footprint pattern analysis during neuropathic nociception induced after weekly intraperitoneal injection of cisplatin (Cis) at 3.0 mg/kg (Cis-3) for four consecutive weeks in rats. The measured parameter was expressed as the overlap distance between the forepaw and hind paw placements, 1 h post treatment. (DOCX 20 kb)

## Data Availability

All data that support the findings of this study are available from the corresponding author upon reasonable request.

## Abbreviations

CG: Control gel
CGRP: Calcitonin gene-related peptide
CIPN: Chemotherapy induced peripheral neuropathy
Cis-3: Cisplatin
GABA_A_: GABAA receptor
GBP-10%: Topical gabapentin
GBP-75: Systemic gabapentin
NMDA: N-methyl-D-aspartate
NO: Nitric oxide
PWL: Paw withdrawal latency
PWT: Paw withdrawal threshold
TRP: Transient receptor potential channel
TRPVI: Transient receptor potential cation channel subfamily V member 1
