# Supplementary material for: Efficacy of a topical gabapentin gel in a cisplatin paradigm of chemotherapy-induced peripheral neuropathy
Source: BMC Pharmacol Toxicol. 2019 Aug 28;20:51. doi: 10.1186/s40360-019-0329-3 (PMC6714310; doi:10.1186/s40360-019-0329-3)
Supplement: Supplementary file 1 — Table S1. Effect of per se treated topical gabapentin 10% gel (GBP-10%), topical control gel (CG) and systemic gabapentin at 75 mg/kg (GBP-75, i.p.) during the expression of cisplatin-induced static allodynia [diminished von Frey filament threshold pressure (paw withdrawal threshold; PWT in g)] in hind paws after weekly intraperitoneal injection of cisplatin at 3.0 mg/kg (Cis-3) for four consecutive weeks in rats. Table S2. Effect of per se treated topical gabapentin 10% gel (GBP-10%), topical control gel (CG) and systemic gabapentin at 75 mg/kg (GBP-75, i.p.) during the expression of cisplatin-induced heat hypoalgesia [increased nociceptive response latency to heat stimulus in the hot-plate paradigm (paw withdrawal latency; PWL in s)] in bilateral hind paws after weekly intraperitoneal injection of cisplatin at 3.0 mg/kg (Cis-3) for four consecutive weeks in rats. Table S3. Effect of per se treated topical gabapentin 10% gel (GBP-10%), topical control gel (CG) and systemic gabapentin at 75 mg/kg (GBP-75, i.p.) on rotarod performance during neuropathic nociception induced after weekly intraperitoneal injection of cisplatin (Cis) at 3.0 mg/kg (Cis-3) for four consecutive weeks in rats. Table S4. Effect of per se treated topical gabapentin 10% gel (GBP-10%), topical control gel (CG) and systemic gabapentin at 75 mg/kg (GBP-75, i.p.) on footprint pattern analysis during neuropathic nociception induced after weekly intraperitoneal injection of cisplatin (Cis) at 3.0 mg/kg (Cis-3) for four consecutive weeks in rats. The measured parameter was expressed as the overlap distance between the forepaw and hind paw placements, 1 h post treatment. (DOCX 20 kb) [file 40360_2019_329_MOESM1_ESM.docx]

**SUPPLEMENTARY MATERIAL**

**Efficacy of a topical gabapentin gel in a cisplatin paradigm of chemotherapy-induced peripheral neuropathy**

Muhammad Shahid^1,2^, Fazal Subhan^2,3*^, Nisar Ahmad^2,4^, Robert D. E. Sewell^5*^

**Affiliations**

^1^Department of Pharmacy, Sarhad University of Science and Information Technology, Peshawar, Pakistan

^2^Department of Pharmacy, University of Peshawar, Peshawar-25120, Pakistan

^3^Department of Pharmacy, CECOS University, Peshawar, Pakistan

^4^Department of Pharmacy, Abasyn University, Peshawar, Pakistan

^5^Cardiff School of Pharmacy and Pharmaceutical Sciences, Cardiff University, Cardiff CF10 3NU, UK

**Email addresses**

Muhammad Shahid: shahidsalim_2002@hotmail.com

Fazal Subhan: fazal_subhan@uop.edu.pk

Nisar Ahmad: nisarahmadsatal@yahoo.com

Robert D. E. Sewell: sewell@cardiff.ac.uk

**Running head:** Topical gabapentin for chemotherapy-induced neuropathy

***Correspondence**

Dr. Fazal Subhan

Professor and Chairman

Department of Pharmacy

CECOS University

Hayatabad, Phase 6, Peshawar

Khyber Pakhtunkhwa, Pakistan

Email: fazal_subhan@uop.edu.pk

subhan@cecos.edu.pk

fsubhan2000@yahoo.com

Professor Robert D. E. Sewell

Cardiff School of Pharmacy and Pharmaceutical Sciences

Cardiff University

Cardiff CF10 3NU, United Kingdom

Email: sewell@cardiff.ac.uk

**Table S1:** Effect of *per se* treated topical gabapentin 10% gel (GBP-10%), topical control gel (CG) and systemic gabapentin at 75 mg/kg (GBP-75, i.p.) during the expression of cisplatin-induced static allodynia [diminished von Frey filament threshold pressure (paw withdrawal threshold; PWT in g)] in hind paws after weekly intraperitoneal injection of cisplatin at 3.0 mg/kg (Cis-3) for four consecutive weeks in rats

| **Days/Groups** | **GBP-10%** | **GBP-75** | **CG** |
| --- | --- | --- | --- |
| **Day 0** | 13.93 ± 0.36 | 14.68 ± 0.16 | 14.55 ± 0.24 |
| **Day 7** | 14.26 ± 0.26 | 14.33 ± 0.23 | 14.58 ± 0.29 |
| **Day 14** | 14.21 ± 0.28 | 14.61 ± 0.22 | 14.21 ± 0.31 |
| **Day 21** | 14.65 ± 0.26 | 14.36 ± 0.41 | 14.53 ± 0.31 |
| **Day 28** | 14.36 ± 0.31 | 14.26 ± 0.25 | 14.21 ± 0.31 |

Each symbol represents the mean PWT in g ± SEM, *n* = 6 rats per group.

**Table S2:** Effect of *per se* treated topical gabapentin 10% gel (GBP-10%), topical control gel (CG) and systemic gabapentin at 75 mg/kg (GBP-75, i.p.) during the expression of cisplatin-induced heat hypoalgesia [increased nociceptive response latency to heat stimulus in the hot-plate paradigm (paw withdrawal latency; PWL in s)] in bilateral hind paws after weekly intraperitoneal injection of cisplatin at 3.0 mg/kg (Cis-3) for four consecutive weeks in rats

| **Days/Groups** | **GBP-10%** | **GBP-75** | **CG** |
| --- | --- | --- | --- |
| **Day 0** | 16.50 ± 0.76 | 16.66 ± 0.49 | 16.50 ± 0.67 |
| **Day 7** | 15.83 ± 0.91 | 19.66 ± 0.84 | 16.83 ± 0.61 |
| **Day 14** | 16.16 ± 0.47 | 19.66 ± 1.21 | 16.66 ± 0.76 |
| **Day 21** | 17.00 ± 0.85 | 20.83 ± 1.01 | 16.33 ± 0.76 |
| **Day 28** | 17.50 ± 0.42 | 20.66 ± 1.08 | 17.66 ± 0.84 |

Each symbol represents the mean PWL in s ± SEM, *n* = 6 rats per group.

**Table S3:** Effect of *per se* treated topical gabapentin 10% gel (GBP-10%), topical control gel (CG) and systemic gabapentin at 75 mg/kg (GBP-75, i.p.) on rotarod performance during neuropathic nociception induced after weekly intraperitoneal injection of cisplatin (Cis) at 3.0 mg/kg (Cis-3) for four consecutive weeks in rats

| **Days/Groups** | **GBP-10%** | **GBP-75** | **CG** |
| --- | --- | --- | --- |
| **Day 0** | 198.000 ± 5.961 | 202.166 ± 3.876 | 199.000 ± 4.802 |
| **Day 7** | 200.833 ± 6.257 | 106.500 ± 13.55 | 197.500 ± 4.978 |
| **Day 14** | 198.833 ± 4.657 | 109.500 ± 8.345 | 194.500 ± 6.443 |
| **Day 21** | 203.833 ± 6.031 | 104.166 ± 13.22 | 199.500 ± 6.576 |
| **Day 28** | 199.333 ± 5.959 | 106.666 ± 11.91 | 197.166 ± 4.061 |

Each symbol represents the mean endurance latency in s ± SEM, *n* = 6 rats per group.

**Table S4:** Effect of *per se* treated topical gabapentin 10% gel (GBP-10%), topical control gel (CG) and systemic gabapentin at 75 mg/kg (GBP-75, i.p.) on footprint pattern analysis during neuropathic nociception induced after weekly intraperitoneal injection of cisplatin (Cis) at 3.0 mg/kg (Cis-3) for four consecutive weeks in rats. The measured parameter was expressed as the overlap distance between the forepaw and hind paw placements, 1 h post treatment.

| **Days/Groups** | **GBP-10%** | **GBP-75** | **CG** |
| --- | --- | --- | --- |
| **Day 0** | 0.959 ± 0.022 | 0.961 ± 0.027 | 0.968 ± 0.029 |
| **Day 7** | 0.953 ± 0.028 | 1.413 ± 0.075 | 0.973 ± 0.029 |
| **Day 14** | 0.972 ± 0.028 | 1.394 ± 0.067 | 0.951 ± 0.027 |
| **Day 21** | 0.961 ± 0.031 | 1.417 ± 0.071 | 0.972 ± 0.021 |
| **Day 28** | 0.961 ± 0.024 | 1.425 ± 0.049 | 0.975 ± 0.029 |

Each symbol represents the mean paw overlap in cm ± SEM, *n* = 6 rats per group.
